# The TRUST Study—TRansition US Together: Evaluating the Impact of a Parent- and Adolescent-Centered Transition Toolkit on Transition Readiness in Patients with Juvenile Idiopathic Arthritis and Childhood-Onset Systemic Lupus Erythematosus †

**DOI:** 10.3390/children11070881

**Published:** 2024-07-20

**Authors:** Simran Heera, Karen Beattie, Zubin Punthakee, Briano DiRezze, Julie Herrington, Tania Cellucci, Liane Heale, Mark Matsos, Jan Willem Gorter, Michelle Batthish

**Affiliations:** 1 School of Rehabilitation Science, Faculty of Health Sciences, McMaster University, Hamilton, ON L8S 1C7, Canada; heeras@mcmaster.ca (S.H.); direzzbm@mcmaster.ca (B.D.); jherring@mcmaster.ca (J.H.); 2 Department of Pediatrics, McMaster University, Hamilton, ON L8S 4L8, Canada; beattik@mcmaster.ca (K.B.); celluct@mcmaster.ca (T.C.); healel@mcmaster.ca (L.H.); gorter@mcmaster.ca (J.W.G.); 3 Department of Medicine, McMaster University, Hamilton, ON L8N 3Z5, Canadamatsosmp@mcmaster.ca (M.M.); 4 CanChild Centre for Childhood Disability Research, McMaster University, Hamilton, ON L8S 1C7, Canada

**Keywords:** adolescent/young adult, AYA, cSLE, childhood-onset systemic lupus erythematosus, JIA, juvenile idiopathic arthritis, transition

## Abstract

Objective: Adolescents with chronic rheumatic disease must increasingly take on more responsibility for disease management from parents as they transition from pediatric to adult care. Yet, there are limited resources to inform and support parents about transition. Here, we evaluate the impact of a Transition Toolkit, geared towards parents and adolescents, on transition readiness, and explore the potential impact of parent–adolescent communication. Methods: A prospective cohort study of youths aged 14–18 years old and their parents was performed. Participant demographics, disease characteristics, transition readiness scores (Transition-Q, max 100), and parent–adolescent communication scores (PACS, max 100) were collected at enrollment (when the Transition Toolkit was shared with adolescents and their parents. Generalized estimating equation (GEE) analyses determined the influence of the Toolkit on transition readiness and explored the role of parent–adolescent communication quality. Subgroup analyses were conducted by sex. Results: A total of 21 patients were included; 19 completed one post-intervention Transition-Q and 16 completed two. Transition-Q scores increased over time and the rate of increase doubled after the Toolkit was shared (β = 7.8, *p* < 0.05, and β = 15.5, *p* < 0.05, respectively). Conclusion: Transition readiness improved at each follow-up, the greatest increase was seen after the Toolkit was shared. Parent–adolescent communication quality did not appear to impact changes in transition readiness.

## 1. Introduction

Juvenile idiopathic arthritis (JIA) and childhood-onset systemic lupus erythematosus (cSLE) are common, chronic pediatric rheumatic disorders. Adolescent patients with JIA will need ongoing care into adulthood as more than 45% continue to experience active disease 18 years post-disease onset, due to ongoing active arthritis, articular damage, and extra-articular manifestations [1,2]. SLE is a multisystemic disorder characterized by the inflammation of blood vessels and connective tissue, and the presence of autoantibodies in bloodwork [3]. Although it is more commonly diagnosed in adulthood, those diagnosed in childhood experience a higher burden of disease [3]. This leads to poor quality of life and has been shown to affect 5- and 10-year morbidity rates [4].

The care of adolescent and young adult (AYA) patients with rheumatic disease has greatly changed over the last few decades given the advent of biologic therapies allowing for improved health outcomes and lifespans [5]. During adolescence and young adulthood, individuals experience multiple life transitions across health, psychological, educational, and social domains. It is critical to consider these inter-relationships when creating effective transition programs to holistically support AYAs to improve the chances of successful transition and health outcomes [5]. Key skills necessary for independent disease management such as executive functioning, planning, and decision making, do not fully develop and mature until young adulthood. Given that adolescents typically transfer to adult care around the age of 18, there is a critical need to provide developmentally appropriate skill training and opportunities to practice such skills prior to and even after the transition to adult care [5].

While a successful healthcare transition has been defined in different ways using different variables (e.g., regular follow-ups within a specified timeframe, treatment adherence, acquisition of transition knowledge, etc.), the main goals are for adolescents to be able to care for themselves, manage their own care, and remain engaged with the healthcare system [6,7]. The Society of Adolescent Health and Medicine defines healthcare transition as “the purposeful, planned movement of adolescents and young adults with chronic physical and medical conditions from child-centered to adult-oriented healthcare systems” [8]. Despite transition programs focusing on skill development to improve adherence and self-management, they are generally not well integrated into pediatric and adult healthcare systems [9,10]. Standardizing the transition process would help to ensure that a minimal standard of care is provided to all patients to account for the variability in the delivery, structure, target population, and the healthcare team members involved of the transition programs [7].

While the SMART model (the Socio-ecological Model of AYA Readiness for Transition) describes the patient and the provider/system as two key components to transition, the third, and less well recognized and studied, is the parent [6]. The model identifies socio-ecological, socio-demographics, and personal variables that are modifiable with an intervention to improve transition readiness in adolescents, and highlights the influence of the dynamic relationships between patient, parent, and provider/system on transition [6]. Transition research on patient–parent relationships in AYA with rheumatologic diagnoses focus on identifying healthcare needs and gaps in transition education, barriers to transition, the influence of parental involvement on patient self-management skills, but little to no research exists on the influence of dyadic parent–adolescent relationships on transition readiness [11,12].

As adolescents age, there is an inherent shifting of responsibility of disease management from parent to patient [13]. Yet, this shift and its impact is not well explored and many parents report that they do not receive adequate guidance to help their child transition to adult care [14]. There is little information on how parent-focused resources used in healthcare practice impact transition readiness, leaving a gap in the literature on how to best support parents themselves, and how to support them in their journey with their child [13]. To begin to investigate these gaps, the TRansition US Together (TRUST) study aimed to (i) determine the impact of a parent- and adolescent-centered Transition Toolkit on self-management skills and transition readiness in youth 14 to 18 years old with JIA or SLE, (ii) explore the influence of the parent–adolescent communication on adolescent’s transition readiness, and (iii) obtain feedback the use of the Toolkit during transition.

## 2. Materials and Methods

### 2.1. Setting

This prospective cohort study recruited participants from the Pediatric Rheumatology Transition Clinic at McMaster Children’s Hospital (MCH), which includes a multidisciplinary team of the pediatric and adult rheumatologist, nurses, child life specialists, and an Advanced Physiotherapist Practitioner who specializes in arthritis care. Adolescents typically begin visits in the Transition clinic around age 14 and continue until their last visit around age 18 when they are transferred to adult care.

### 2.2. Participants and Data Collection

Participants were recruited from the MCH Transition clinic from May 2020 to September 2023 if they were (i) 14 to 18 years old and (ii) diagnosed with JIA or cSLE as per the International League of Associations for Rheumatology (ILAR) and American College of Rheumatology (ACR) criteria, respectively [15,16]. As part of routine clinical documentation, adolescents are asked to complete the Transition-Q (see Section 2.3) at every visit. To be eligible for this study, participants were required to completed at least 2 Transition-Qs prior to enrolment. Participants were excluded if had special healthcare needs that would prevent them from transitioning to adult care independently, did not read or understand English, or if they did not have a parent present at time of consent.

Approval for this study, number 12653, was approved by Hamilton integrated Research Ethics Board (HiREB). After consent was obtained, adolescents and their parent were given the Transition Toolkit and were asked to the complete the appropriate Parent–Adolescent Communication Scale (PACS) questionnaires (see Section 2.4). Participant demographics and patient disease characteristics were collected at study enrolment. At their regularly scheduled follow-up appointments in Transition Clinic, which can range from 3 to 12 months depending on disease activity and care needs, a third and possibly fourth Transition-Q score was obtained. From January to September 2023, a feedback questionnaire was given to adolescents and parents at their rheumatology follow-up visit to reflect on the Toolkit and comment on how useful they found it, frequency of use, if they discussed it with their parent/child, and suggestions on how to improve Toolkit delivery and utilization.

### 2.3. Transition Toolkit

The Transition Toolkit is an educational resource that is informed by research evidence on healthcare self-management, and was created in collaboration with MCH healthcare providers, researchers, adolescents, and parents [13]. The Toolkit includes 3 components: a parent-focused transition pamphlet, a patient-focused transition roadmap, and tip sheets embedded in both to improve self-management skills (https://www.canchild.ca/en/research-in-practice/current-studies/trust-study; accessed on 14 May 2024) [17,18]. The transition roadmap is built on 5 domains of transition readiness: (1) self-advocacy, (2) medication management, (3) overall health and safety, (4) lifestyle and behaviors, and (5) future planning [13,17,18]. The pamphlet and roadmap are a source of information and a guide for discussion between adolescents and parents to develop goals that they can work towards to become more independent and improve self-management skills.

### 2.4. Outcome Measures

#### 2.4.1. Demographic and Disease Characteristics

Demographic characteristics collected at baseline included age at diagnosis, sex, age, height, weight, and education stream. Parent demographics including age group, relationship with adolescent, and home environment (dual/single parent homes and members within the household) were also reported by the parent. Four measures of disease activity were collected: (i) clinical Juvenile Arthritis Disease Activity Score (cJADAS), which combines physician and patient global disease activity scores with the active joint count to obtain a score out of 30, where higher values indicate higher disease activity [19]; (ii) Childhood Health Assessment Questionnaire (CHAQ), which measures the physical functioning of children with arthritis, scored from 0 (no disability) to 3 (severe disability) [20]; (iii) Pediatric Quality of Life Inventory (PedsQL), which measures the quality of life across physical, emotional, social, and school functioning domains on a scale of 0–100, with higher scores indicating greater quality of life [21]; (iv) pain scores using the numerical rating scale ranging from 0 (no pain) to 10 (severe pain) [22].

#### 2.4.2. Transition Readiness

To address the primary and secondary objectives, transition readiness was captured using the Transition-Q, a 14-item, validated transition readiness assessment tool for adolescents with chronic disease [13,23]. The Transition-Q is scored on a scale of 0–100, with higher scores reflecting greater self-management skills. For each question, the adolescent is asked to indicate the frequency at which they do the task (never, sometimes, always). Questions are posed in order from the most simple (requiring less independence) to more complex (requiring a high level of independence). Once the questionnaire is completed, a Child Life Specialist or nurse reviews the adolescent’s responses and identifies the simplest questions (lowest number) to which the adolescent has not answered “always”. Following a discussion with the adolescent, this question is then identified as a goal to work towards for the next visit. This plan is followed throughout subsequent clinic visits. Each adolescent may set 1–2 goals at each appointment. The Transition-Q was found to be a reliable and valid tool. However, it has not been validated to detect change in transition readiness scores over time or beyond ages 12 to 18 years old [23]. Transition readiness scores were scheduled to be collected twice before the intervention and twice afterwards.

#### 2.4.3. Parent–Adolescent Communication Scale (PACS)

To address the second objective, communication quality between parent and child was assessed to determine its potential influence on the child’s independence and self-management skills [24]. The PACS (max 100) was administered from both the child and parent, with each given the option to complete the survey confidentially in separate spaces. This validated, 20-item tool has two subscales to evaluate the openness and problems in communication between parent and child. Both subscales are scored from 1 to 5; however, the openness subscale values higher scores, and the problem in communication subscale values lower scores. The PACS questionnaire does not have a specific cut off to define what a ‘good score’ is. Overall, higher scores indicate greater quality of communication between parent and child. To our knowledge, the TRUST study is the first to use the PACS questionnaire to explore parent–adolescent communication within rheumatology.

#### 2.4.4. Participant Feedback on Transition Toolkit Use

To address the third objective, we surveyed adolescents and parents during Transition Clinic appointments about their perspectives and experiences of the Toolkit’s organization, content, applicability, and utility. The survey prompted adolescents and parents to reflect on whether they discussed the content of the Toolkit with each other, how frequently it was used, and how helpful it was. Participants were asked to rate its usefulness on a Likert scale of 1 to 5 (where higher scores reflect greater usefulness) and respond to an open-ended question to describe any changes they would make to the Toolkit to improve its delivery and implementation.

### 2.5. Statistical Analyses

Anticipating the percentage of participants that would be seen in Transition Clinic over the recruitment period, the original goal was to collect data from 69 patient–parent dyads. This calculation was performed while anticipating the percentage of participants that would be seen in Transition Clinic during the recruitment period that meet inclusion criteria and a conservative estimate of 90% participants consenting. This sample size was not reached due to this study’s limited time frame and impacts from COVID-19. All data were collected prior to September 2023. A total of 21 participants were included in this study.

To describe the patient, parent, and disease characteristics for the study population, means and standard deviations were determined from normal data, and medians and interquartile range (IQR) were determined from non-normal data for continuous variables, where appropriate, and frequencies and proportions for categorical variables.

The significance of changes in Transition-Q scores before and after the Toolkit was shared with participants was determined using generalized estimating equation (GEE) analyses [25,26,27]. A linear GEE regression model was used to account for the correlation between Transition-Q scores from multiple time points for each participant with an unstructured correlation matrix, while controlling for age and sex. The model examined the significance of changes in Transition-Q scores over time throughout the study period and after the intervention, while evaluating for potential association with sex and age of the adolescent.

Means and standard deviations of PACS scores were determined for parent and adolescent PACS scores and were also compared by sex. To determine if adolescent-reported communication quality with their parent influenced Transition-Q scores, the GEE analysis was run with adolescent PACS scores and sex included in the model. Normality of adolescent PACS scores was tested using the Shapiro–Wilk test. Homogeneity of variance and the 95% confidence interval (CI) were calculated using the variance ratio test. The Wilcoxon signed rank test for smaller sample sizes was used for adolescent PACS scores as they were not normally distributed.

Data analyses were performed using STATA software, version 18, for Mac OS.

### 2.6. Questionnaire Analysis

Means and proportions from self-reported ratings (using the Likert scale) on the usefulness of the Toolkit were calculated. Feedback from responses to the open-ended questions on the questionnaire were analyzed using inductive qualitative content analysis [28]. Coding was conducted by the lead author, who had been familiarized with the data through repeated rereading and identifying frequently used words. The lead author noted down words or phrases in responses that were frequently used or had value and labels were generated to summarize their meaning. Once coding was complete, the lead author inspected the codes for similarities and differences and then grouped them into categories. Data were reviewed multiple times to confirm that the selected categories were representative. Quotes were used to illustrate and support the analysis; these were included verbatim with no changes to spelling or grammatical errors.

## 3. Results

### 3.1. Study Participants

**Figure 1:**
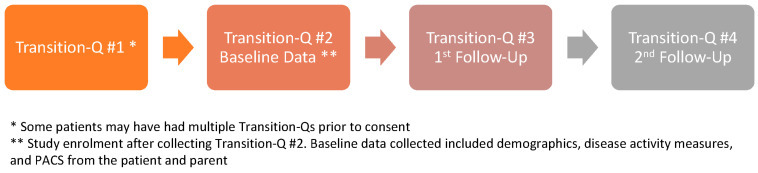
Flow of study timeline.

A total of 21 patient–parent dyads consented to participate; of these, 16 (76%) were diagnosed with JIA and 5 (24%) with SLE. Participant demographics and disease characteristics obtained at baseline are summarized in Table 1. Of the 21 participants included, 19 completed the first post-intervention Transition-Q assessment, and 16 completed the second (Figure 1). All 21 participants completed the PACS questionnaire, and one parent did not. The feedback questionnaire was distributed to adolescents and parents who attended follow-up appointments between January and September 2023, yielding responses from nine adolescents and six parents.

### 3.2. Impact of the Transition Toolkit on Transition Readiness

**Figure 2:**
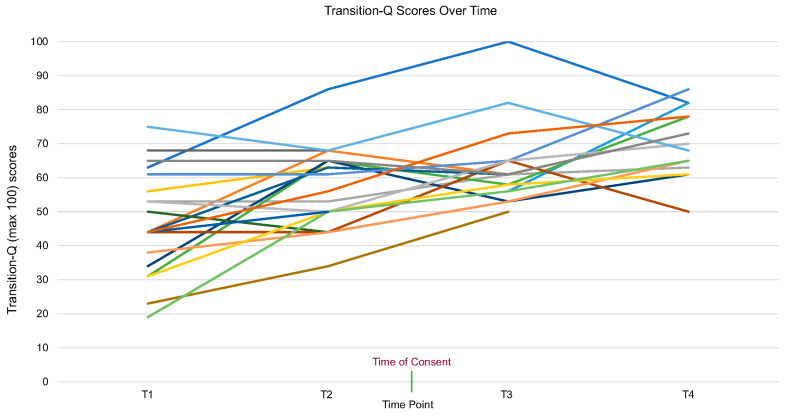
Changes in Transition-Q scores over time for each participant (each line represents individual patient participants).

**Figure 3:**
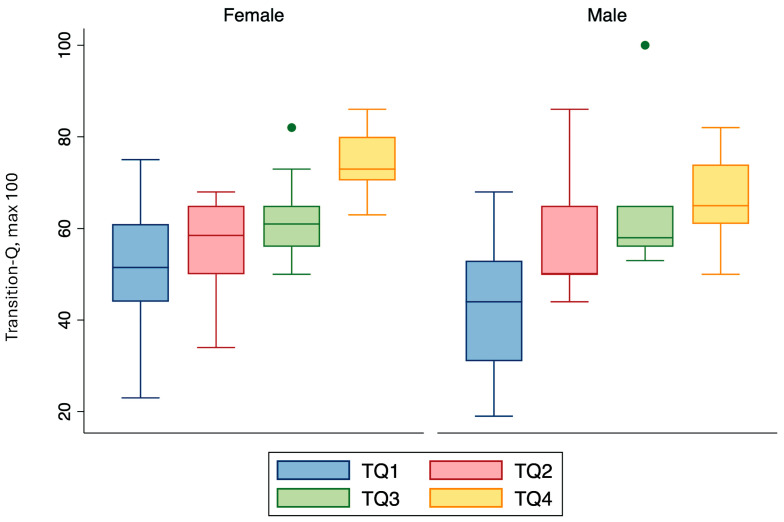
Transition-Q scores at each time point by sex. Outliers are shown as single dots.

Transition-Q scores of each participant were collected twice before and twice after the Transition Toolkit intervention was delivered. Mean (SD) Transition-Q scores increased over time, with a mean score of 46.9 (14.7), 57.0 (11.8), 62.7 (11.7), and 70.5 (9.5), at each time point (Figure 2). When observing changes in Transition-Q by sex, both males and females demonstrated similar improvement over time (Figure 3). The rate of change in transition readiness scores from the first to the fourth time point was significant (β = 7.8, *p* < 0.05, 95% CI = 6.3, 9.3). Transition-Q scores just prior to and after the Transition Toolkit intervention increased by 15 points (β = 15.5, *p* < 0.05, 95% CI = 12.8, 18.3). After controlling for age and sex, results from the GEE analysis showed little change in the regression coefficient (β = 8.1, *p* < 0.05, 95% CI = 6.8, 9.3 over entire study duration and β = 14.3, *p* < 0.05, 95% CI = 11.8, 16.7 for before versus after Toolkit intervention).

### 3.3. Influence of Parent–Adolescent Relationships on Transition Readiness

**Figure 4:**
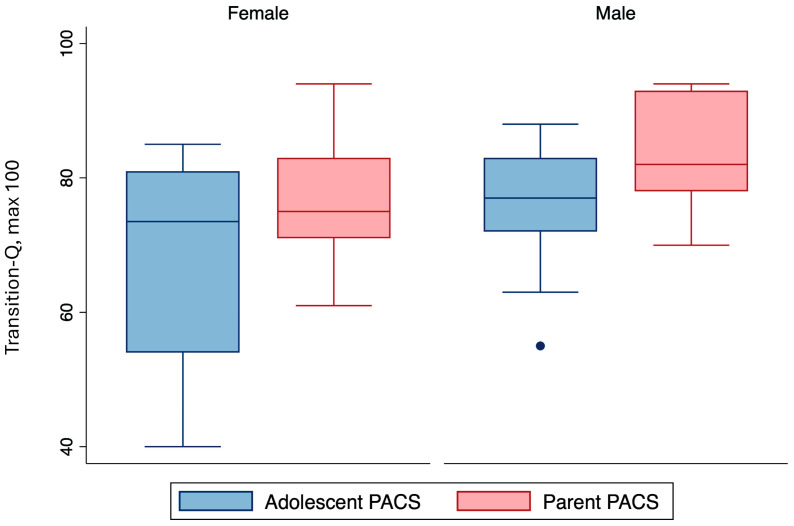
Parent and adolescent PACS scores by sex. Outliers are shown as single dots.

Of the 21 participants, PACS score data were missing from one parent. Parent-reported communication quality scores (mean = 80.7, SD = 9.6) appeared higher than their child’s (mean = 72.3, SD = 13.0). Quality of communication between parent and adolescent when reported by males (mean = 75.7, SD = 9.9) appeared slightly higher than females (mean = 68.6, SD = 15.4), although the difference was not significant *p* = 0.4 (Figure 4). When comparing adolescent PACS and Transition-Q scores at baseline by sex, the GEE analysis showed that PACS scores did not affect Transition-Q scores.

### 3.4. Feedback on Transition Toolkit Effectiveness on Transition Readiness

Nine adolescent and six parent responses were analyzed using qualitative content analysis to identify the main theme from the Toolkit feedback. Of those who responded, 67% of participants found the Toolkit somewhat or slightly useful. Six participants (two adolescents, four parents) responded to the open-ended question by commenting on how to better share the Toolkit’s content and improve ease of access. Based on adolescent and parent responses, the main theme that emerged was that the lack of review of the toolkit content at follow-up appointments resulted in them forgetting the content and not applying what they read (Table 2). In total, two categories of data were identified within the main theme—misplacing or forgetting the Toolkit, and reminders may be helpful.

Respondents reported difficulty in recalling the content of the Transition Toolkit given that they did not review it with their adolescent/parent after it was initially provided to them. Many explained that they either misplaced the Toolkit or forgot the content simply because it was not reviewed in follow-up clinic appointments. One parent noted that while the Toolkit was discussed at the appointment it was given and on their way home with their adolescent, they admit that “it got buried in my desk”, causing them to forget the Toolkit’s content. Other respondents shared similar answers and suggested reminders may be helpful in remembering to reference the Toolkit more often.

## 4. Discussion

The TRUST study aimed to determine the impact of a Transition Toolkit and the role of the parent–adolescent relationship on transition readiness. We report that transition readiness increased significantly after the Transition Toolkit was shared with participants and their parents. The quality of communication between adolescent and parent did not influence transition readiness. The feedback from the questionnaire suggests that the use of the Transition Toolkit could be improved if it was reviewed regularly in the clinic setting.

### 4.1. Impact of Transition Toolkit on Transition Readiness

The findings from this study demonstrate that adolescents’ level of transition readiness, measured with the Transition-Q, improved at each follow-up with identifying areas for improvement and setting goals. This finding is supported by the current literature, which suggests that age-related improvement in transition readiness is a result of developing skills from life experiences that are relevant to healthcare management [29]. A previous study found that in patients with JIA or cSLE, transition readiness measured with the Transition-Q was higher in older adolescents, which may be associated with greater exposure to in-clinic transition resources and continuous involvement in discussions about transition [30]. To our knowledge, longitudinal assessments of transition readiness in patients with rheumatic disease have not been explored.

While a majority of study participants’ Transition-Q scores increased with time (71%), positive increments in transition readiness scores were not consistent for all participants. Age alone cannot predict adolescents’ level of preparedness to transfer to adult care, as skill acquisition is not a linear process, nor is it the same across individuals [5]. This suggests that other mediating factors may be influencing transition readiness, such as transition knowledge, relationships with the healthcare team and parent(s), goals and expectations, etc. [6]. Notably, the greatest improvement of transition readiness in this study was seen from the second to third time point representing the period immediately after the implementation of the Toolkit with adolescents and their parent. Subgroup analyses showed similar improvement in transition readiness in males and females over the study duration.

### 4.2. Influence of Parent–Adolescent Relationships on Transition Readiness

Within this cohort, parent-reported PACS scores were greater than adolescents’. Importantly, there was a large amount of variability in adolescent PACS scores compared to parents. In addition, female adolescents had significantly greater variables in PACS scores compared to male adolescents. Transition readiness did not seem to be affected by the communication quality between adolescent and parent. However, other aspects of the parent–adolescent relationship (acceptance, emotional availability, attachment, etc.) were not considered or measured with the PACS. The current literature on transition shows a limited understanding of the relationship dynamics within families and between parent and child based on sex, the burden of healthcare responsibility, and assumed gender-based social roles from a transition lens [31]. Some studies note that greater parental involvement is associated with greater transition readiness. Suggesting that developmentally appropriate parental involvement allows adolescents to foster and practice skills for disease management as they advance towards adult care. However, others note that protective parents may inhibit adolescent independence, self-efficacy, and communication skills with providers. This in turn leads parents to underestimate adolescent’s transition readiness, express hesitancy to transfer healthcare responsibility to the adolescent, and experience a lower quality of life [5,31,32,33].

Most participating parents in this study were mothers, aligning with the literature describing how mothers traditionally take on the role of caretakers. One participating father noted that discussion of healthcare and disease management did not extend outside of the mother–child dyad, and thus, he was not informed of the child’s healthcare and needs. Further, transition research investigating parent–adolescent relationships is limited within rheumatology but supports the finding that mothers mainly adopt responsibility of adolescent healthcare management and experience more intimate relationships compared to fathers [31].

### 4.3. Feedback on Transition Toolkit Effectiveness on Transition Readiness

Responses from adolescents and parents on the content and use of the Transition Toolkit are essential in standardizing future transition programs and to better understand participant needs for greater dissemination of transition care. Based on the results from surveys, the Transition Toolkit may be a helpful tool in mapping out an adolescent’s journey through pediatric care as they prepare for the transfer to adult rheumatology. Feedback on the usefulness of the Transition Toolkit poses a risk for recall bias. The time elapsed between the initial Toolkit introduction and survey distribution was different for each participant, prompting participants to reflect on an event that occurred possibly many months ago. As a result, answers from adolescents and parents were highly variable and subject to memory and response bias. While only a few participants responded, common themes arose including reminders, reviewing the Toolkit at follow-up, or having a digital platform for the Toolkit to improve its utilization. Greater uptake and delivery of the Toolkit is necessary as many respondents noted not reviewing the Toolkit after consent. Integrating the tailored Transition Toolkit and sharing it with patient–parent dyads when developmentally appropriate as part of clinical care may be valuable in reminding both parents and adolescents of its content and applicability. In addition, in-depth discussions with adolescents and parents on transition, life events, and goals may be insightful into participants’ daily lives to pinpoint what may be positively or negatively influencing transition readiness.

### 4.4. Strengths and Limitations

This study included a small sample size of 21 participants with relatively homogenous demographics. Most participants were from dual-parent homes and enrolled in the study with their mothers. Home environment and parent demographics were similar for most participants, making it difficult to ascertain if the parent–adolescent relationships described in this study would be generalizable to families with differing dynamics and backgrounds. The small sample size of this study suggests that there is insufficient power to extrapolate the findings from this study to the greater pediatric population with rheumatic disease despite statistically significant improvements in Transition-Q scores for most participants. Being mindful that confounders (e.g., time elapsed between each appointment, age at baseline, etc.) were intentionally not controlled for in order to ensure that the study timeline matched clinically, as well as the impact of COVID-19, which impeded recruitment, participants in this study comprise nearly one-fifth of all patients who are followed in the Transition Clinic at MCH. The findings from this study are preliminary as it is limited by recruitment from a single center and its small sample size. Future studies should include a larger, more diverse patient/family population to ensure that findings are more representative of the pediatric population with rheumatic disease.

To our knowledge, the TRUST study is the first to use the PACS questionnaire within rheumatology to explore communication between parent and adolescent. The small variability in PACS scores may explain why it was difficult to determine the potential impact of quality of communication between parent and adolescent on transition readiness. The PACS evaluates the communicative components of parent–adolescent relationships without taking other aspects of the relationship into account. In this current study, PACS scores were only determined once at the time of study enrolment at the time the Transition Toolkit was shared. Thus, possible changes in PACS scores reflecting improved or worsened communication quality between the adolescent and their parent were not determined. Using the PACS longitudinally and exploring other influencing factors may provide a greater understanding of changing parent–adolescent relationships and the impact on adolescent transition readiness.

The Transition Toolkit content seems to be helpful for parents and adolescents during their transition journey. Using feedback from patients and parents, we will integrate the toolkit into each visit in clinic to ensure that topics covered in the toolkit are discussed at each visit. The utilization of the Transition Toolkit by knowledge users (parents and adolescents) may be improved with easier access to its digital version and by setting up reminders integrated within Electronic Health Records to ensure adolescents and parents are reviewing the Toolkit consistently.

### 4.5. Conclusions

Our findings suggest that the Transition Toolkit is a helpful resource for adolescents and parents to navigate the transition from pediatric to adult rheumatology care, as shown by increases in transition readiness scores after as compared to before the introduction of the toolkit. The main limitation of this study is the small sample size, which limited our ability to make robust conclusions from the data. However, these preliminary findings are promising and helpful in leading future investigations. Although the participants have found the Toolkit helpful, they have also noted that it is not frequently referenced by parents and adolescents, thus making it difficult for the Toolkit’s content to be retained. Following the socioecological model of transition readiness, developmentally appropriate care and parental involvement is essential for cultivating skills for independent disease management as adolescents adopt primary responsibility of care from their parent(s) [5]. We suggest implementing the Transition-Q, goal setting, and the Transition Toolkit in a standard fashion in future transition programs and clinical settings to facilitate greater acquisition of healthcare management skills and guide healthcare decision making [34,35,36]. Future studies will investigate the mitigating factors affecting transition readiness, explore the relationship between parent and adolescent further, longitudinally assess communication quality between parent and adolescent, and explore Transition Toolkit implementation clinically and across other disciplines.

## Figures and Tables

**Table 1:** Participant demographics and disease characteristics.

Participant Characteristics	All Participants, n = 21	Females, n = 10	Males, n = 11
Female Participant, n (%)	10 (48%)		
Median Age at Diagnosis (IQR), years	12.0 (25.5)	12.5 (16.0)	11.0 (20.2)
Median Age at Baseline (IQR), years	15.0 (26.5)	14.0 (17.2)	15.0 (21.9)
Mean (SD) cJADAS ^1^, n = 20	1.5 (1.8)	1.3 (2.1)	1.7 (1.6)
Mean (SD) Active Joint Count, n = 20	0.4 (0.8)	0.4 (1.0)	0.4 (0.7)
Mean (SD) CHAQ ^2^, n = 13	0.3 (0.4)	0.3 (0.4)	0.2 (0.5)
Mean (SD) PedsQL ^3^, n = 12	71.1 (28.6)	50.3 (33.7)	81.5 (20.6)
Mean (SD) Pain, n = 20	1.0 (1.4)	0.9 (1.3)	1.0 (1.6)
Student Education with an Academic Stream, n (%)	14 (67%)	8 (80%)	6 (55%)
Dual Parent Home, n (%)	20 (97%)	9 (90%)	11 (100%)
Mom as Participating Parent, n (%)	17 (81%)	9 (90%)	8 (73%)
Participating Parent Over Age of 50, n (%)	8 (38%)	5 (50%)	3 (27%)

^1^ cJADAS—clinical Juvenile Arthritis Disease Activity Score; ^2^ CHAQ—Childhood Health Assessment Questionnaire; ^3^ PedsQL–Pediatric Quality of Life Inventory.

**Table 2:** Main themes from Transition Toolkit feedback.

Theme: Lack of Review at Follow-Up Causes Recall Difficulty
*Corresponding Code: Misplacing or forgetting the Toolkit*
- “We probably talked about it on the way home. I don’t remember what is in the toolkit at the moment. It got buried in my desk.”
- “I looked over it at an appointment, but I did not look through it any further”
*Corresponding Code: Reminders may be helpful*
- “Maybe a reminder we had it because I forgot.”
- “Possibly a digital platform, with reminders.”

## Data Availability

Data are contained within the article and Supplementary Materials.

## Supplementary Materials

Supporting information on the Transition Toolkit can be found at: https://www.canchild.ca/en/research-in-practice/current-studies/trust-study (accessed on 14 May 2024).
